# Digital biomarkers from gaze tests for classification of central and peripheral lesions in acute vestibular syndrome

**DOI:** 10.3389/fneur.2024.1354041

**Published:** 2024-03-26

**Authors:** Benjamin Duvieusart, Terence S. Leung, Nehzat Koohi, Diego Kaski

**Affiliations:** ^1^Department of Medical Physics and Biomedical Engineering, University College London, London, United Kingdom; ^2^SENSE Research Unit, Department of Clinical and Movement Neurosciences, University College London, London, United Kingdom

**Keywords:** acute vestibular syndrome, gaze test, biomarkers, vertigo, nystagmus, machine learning

## Abstract

Acute vestibular syndrome (AVS) is characterised by a sudden vertigo, gait instability, nausea and nystagmus. Accurate and rapid triage of patients with AVS to differentiate central (potentially sinister) from peripheral (usually benign) root causes is a challenge faced across emergency medicine settings. While there exist bedside exams which can reliably differentiate serious cases, they are underused due to clinicians’ general unfamiliarity and low confidence interpreting results. Nystagmus is a fundamental part of AVS and can facilitate triaging, but identification of relevant characteristics requires expertise. This work presents two quantitative digital biomarkers from nystagmus analysis, which capture diagnostically-relevant information. The directionality biomarker evaluates changes in direction to differentiate spontaneous and gaze-evoked (direction-changing) nystagmus, while the intensity differential biomarker describes changes in intensity across eccentric gaze tests. In order to evaluate biomarkers, 24 sets of three gaze tests (left, right, and primary) are analysed. Both novel biomarkers were found to perform well, particularly directionality which was a perfect classifier. Generally, the biomarkers matched or eclipsed the performance of quantitative nystagmus features found in the literature. They also surpassed the performance of a support vector machine classifier trained on the same dataset, which achieved an accuracy of 75%. In conclusion, these biomarkers simplify the diagnostic process for non-specialist clinicians, bridging the gap between emergency care and specialist evaluation, ultimately benefiting patients with AVS.

## 1 Introduction

Dizziness accounts for 4.4 million emergency visits in the US alone (1) and is the most frequent cause of a physician visit for patients over 75 (2). It is estimated 10%–20% of dizzy patients have acute vestibular syndrome (AVS) (3). AVS characterized by a sudden and sustained onset of vertigo, gait instability, nausea, and nystagmus which lasts for over 24 h. The symptoms exhibited by a patient with AVS are associated with a large variety of underlying pathologies, ranging from benign peripheral lesions (e.g., vestibular neuritis) to potentially sinister brain lesions (e.g., stroke) (3). For instance, both cerebellar and brainstem strokes can present as isolated AVS, where the lack of other neurological symptoms complicates their diagnosis (4–6). As such, particularly in emergency settings, rapid and accurate triaging of patients with peripheral and central lesions is key to improving patient outcomes and effective allocation of hospital resources. Unfortunately, many clinicians lack confidence in delivering the relevant diagnostic tests and struggle to accurately interpret the clinical signs (7). This strongly motivates the development of tools which reduce the interpretative burden placed on non-specialist clinicians and help to identify patients who require urgent medical attention. Nystagmus is defined as involuntary eye oscillations characterised by a pathological slow drift of the eye (i.e., slow phase) followed by fast corrective motion (i.e., fast phase); if correctly interpreted it provides significant insight on the cause of the AVS (8). Given it is also the most readily recorded and analysed of the sets of AVS symptoms, this paper focuses on developing biomarkers and features describing horizontal nystagmus across eccentric and primary gaze tests.

While many works have investigated the diagnostic relevance of the nystagmus, there are few which look at extracting quantitative measures for diagnosis or risk stratification. Slow phase velocity (SPV) of the eye rotation is the most commonly extracted measure and has shown good promise (9, 10). Mouelhi et al. (11) have found that combining SPV with additional features such as direction, nystagmus period, and measures of variability provide better results. Calic et al. (12) also investigated quantitative nystagmus features to differentiate between patients with vestibular migraines and vestibular neuritis, but SPV was not found to be statistically significant. A further set of 3 papers by Young et al. (13–15), analysed nystagmus characteristics of healthy controls, patients with vestibular migraine, and patients suffering from Meniere’s disease. The authors noted a significant difference in SPV between vestibular migraine and Meniere’s disease and implemented it in a two variable classification algorithm achieving 95.7% sensitivity and 85.1% specificity (14).

Despite its known diagnostic relevance no studies evaluating changes in nystagmus over gaze directions were found, with all papers above limited by features extracted from a single test in one gaze direction. As such, this work aims to present nystagmus biomarkers which evaluate changes in nystagmus intensity and direction across horizontal eccentric gaze directions to differentiate central and peripheral origins of AVS. These biomarkers reflect the routine clinical assessments of eye movements used by specialists to diagnose patients with acute vertigo. Finally, the biomarkers are compared to single gaze test features, as well as a support vector machine (SVM) to establish a baseline performance of a simple machine learning model.

## 2 Materials and methods

### 2.1 Data

**Figure 1:**
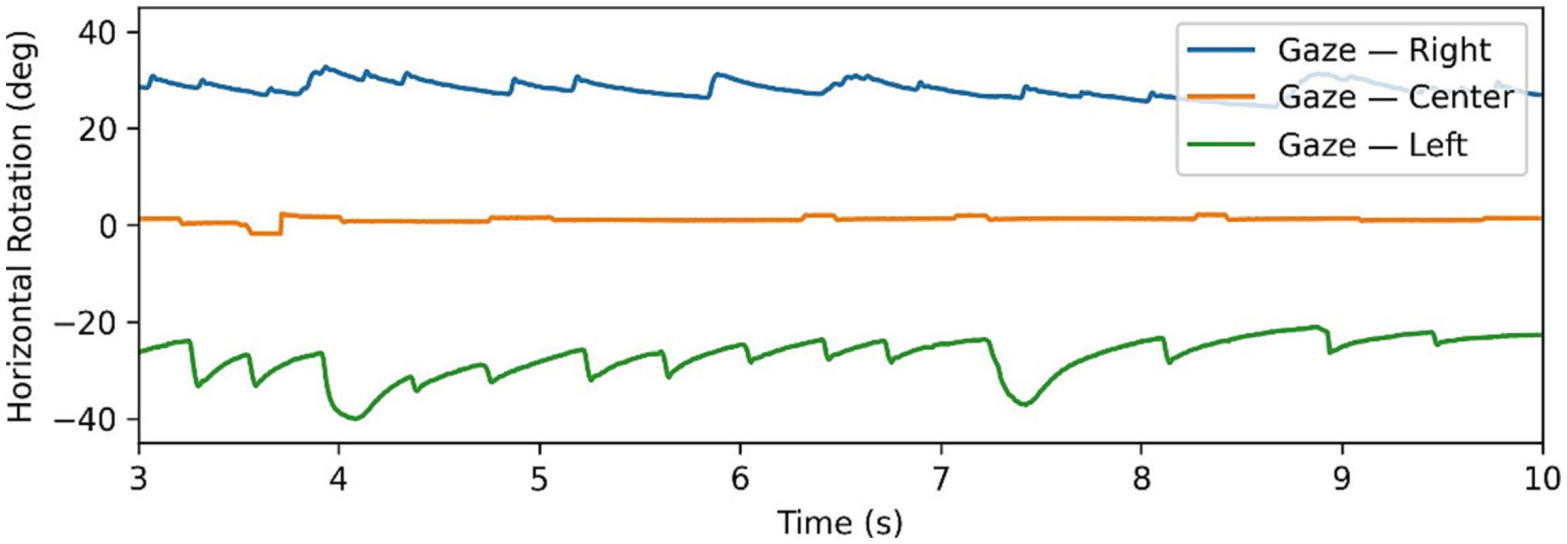
Example of gaze-evoked nystagmus data, with gaze tests time series in all 3 directions superimposed. Note changing directions of fast phases according to direction of gaze.

**Figure 2:**
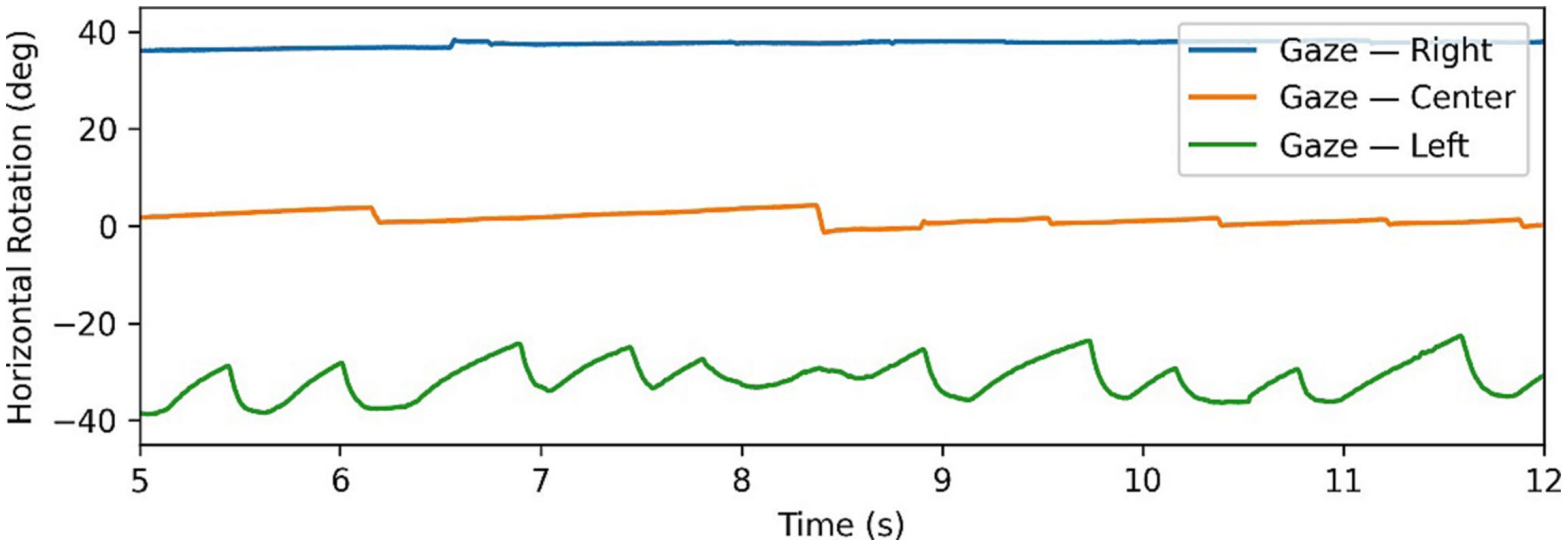
Example of Alexander’s law in nystagmus data, with gaze tests time series in all 3 directions superimposed. Note changing intensity of oscillations depending on direction of gaze.

Nystagmus data was collected from 24 patients (12 peripheral and 12 central) who attended their Neuro-otology appointments in 2022 and 2023 at the National Hospital for Neurology and Neuroscience, London. Patients included in the study were retrospectively selected according to final established diagnosis, inclusion and exclusion criteria. All patients included in the study underwent MRI imaging to confirm final diagnosis as part of the study protocol. Adult patients (>18 years) who had presented with an AVS, including the presence of nystagmus, and evaluated by the acute vertigo service within 2 weeks of symptom onset were included in this study. Exclusion criteria included inability to express consent, a history of pre-existent vertigo in the last 6 months, or alternative neurological or ophthalmological disorders that affect eye movements. Table 1 shows a breakdown of the included patients. For each patient primary (looking straight forward), left, and right gaze tests were collected to evaluate nystagmus behaviour across gaze directions—Figures 1, 2 show these gaze tests for patients with central and peripheral lesions, respectively. Each gaze test consists of recording the eye position in the horizontal and vertical planes for 15–45 s while the patient attempts to maintain a steady gaze direction. Gaze test recordings were acquired with fixation, as this more closely resembles the bedside clinical assessment in emergency settings.

**Table 1:** Patient sex and age distributions, grouped by patient cohort.

	Number	Sex (M/F)	Mean age in years (std dev)
Peripheral lesions	12	9 (75%)/3 (25%)	55.2 (9.7)
Central lesions	12	5 (42%)/7 (58%)	70.0 (11.8)
Total	24	14 (58%)/10 (42%)	62.6 (13.0)

**Figure 3:**
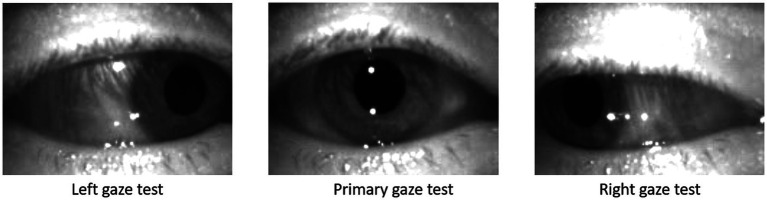
Screen shots from left, primary, and right gaze test recorded on the ICS Impulse (GN Otometics, Taastrup, Denmark) mounted headset using infrared camera.

The gaze tests data were recorded by an experienced audiologist using the ICS Impulse system (GN Otometics, Taastrup, Denmark). The ICS Impulse is a video oculography tool which uses head mounted goggles to record the movements of the patient’s eye. The eye tracking is completed using an infrared camera mounted at close proximity to the right eye as shown in Figure 3. The infrared camera provides improved contrast of the pupil against the iris, thus allowing for accurate tracking of the pupil following calibration steps taken by the clinician. The camera records eye movements at ∼170 frames per second, and will record a position of the eyes in all frames unless the patient is blinking. The position is encoded by vertical and horizontal rotation measured in degrees, generating a time series for each gaze test in a 3×n matrix, corresponding to the timestamp, horizontal rotation, and vertical rotation for the number of recorded frames in the IR video ( n ). Only the raw positional data was extracted from the ICS Impulse, with all further calculations being derived from it—all processing of this data was completed using Python v3.9. Before further analysis, the time series were cropped to remove segments where the gaze was not correctly oriented. This often occurred at the end of a gaze test as patients became nauseous when holding gaze in a direction causing pronounced nystagmus.

### 2.2 Segmentation

To extract quantitative measures describing the nystagmus behaviour, fast and slow phases need to be segmented. As the fast saccades have the strongest signal and best signal-to-noise ratio, they serve as the basis of the segmentation algorithm. This forces the algorithm to assume the presence of nystagmus in the input timeseries, which in turn means the absence of nystagmus can generate unpredictable results. The algorithm is specifically engineered to overcome the two types of noise present in nystagmus traces: high frequency noise caused by errors in eye tracking or mircosaccades (eye jitter), and larger non-nystagmus eye saccades (e.g., refocusing or blinking) which need to be ignored when extracting features. Firstly, the algorithm uses outlier thresholding to locate higher velocity points. Then a Sieve filter is applied to extract contextual information (16) and group individual points into continuous blocks with higher velocities. Once continuous blocks are generated, further filtering leveraging known characteristics of fast phases is used to accurately locate the phase boundaries and differentiate genuine fast phases from noise and other saccades. The remaining points are then classed as being slow phase points, hence providing a final segmentation of the time series into fast phase blocks, slow phase blocks, and erroneous saccades. The segmentation of the nystagmus time series was validated by an expert neurologist for all traces, before proceeding with further processing.

### 2.3 Features and biomarkers

Each individual gaze test was summarised using fundamental features to characterise the eye movements. By themselves, the features contain important descriptive information, but can also be combined to build the proposed biomarkers.

#### 2.3.1 Features

**Figure 4:**
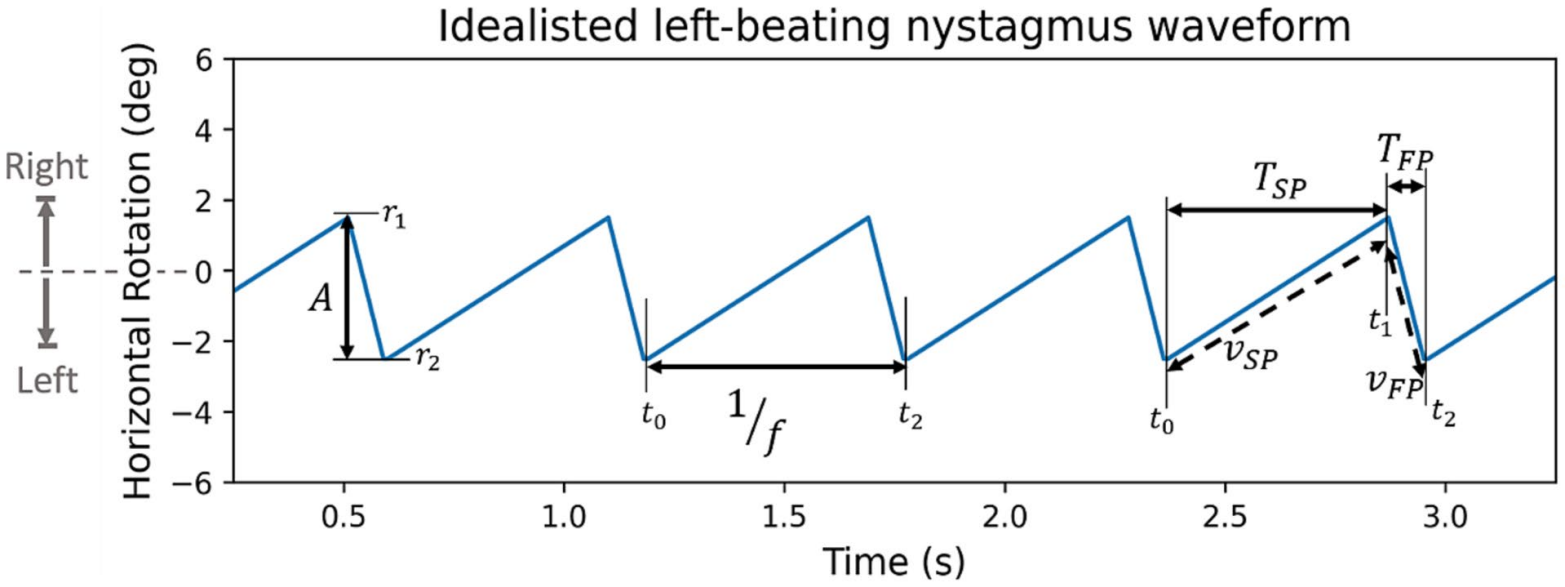
Synthetic left beating nystagmus waveform with annotated features ( A : amplitude, f : frequency, $T_{SP}\&T_{FP}$ : period of slow and fast phase, $v_{SP}\&v_{FP}$ : velocity of slow or fast phase). By convention rightward rotation is positive, and leftward rotation is negative.

Features consider the primary, left, and right gaze tests independently and describe particular aspects of the time series. Figure 4 shows a visual representation of features on an idealised nystagmus waveform.

##### 2.3.1.1 Amplitude

Amplitude, A , is defined as the rotation of the eye during the fast phase (Equation 1)

(1) $$A=|r_{2}-r_{1}|$$

where $r_{\;1}$ and $r_{\;2}$ are the rotation of the eye at the beginning and end of the fast phase. Amplitude of each nystagmus oscillation is found individually, from the resulting distribution we extract average, minimum, maximum, and standard deviation values. Due to the physiological nature of the source data and distribution it is expected to have increased variability and noise. To counteract this, the minimum and maximum values are measured using the 10th and 90th percentiles avoiding non-representative outliers.

##### 2.3.1.2 Frequency

The nystagmus frequency, f , is also found for each individual oscillation first before taking the mean, minimum (10th percentile), maximum (90th percentile), and the standard deviation. The frequency of an individual oscillation is calculated as the reciprocal of its period (Equation 2)

(2) $$f=\frac{1}{t_{2}-t_{1}}$$

where the $t_{1}$ and $t_{2}$ are the timestamp at the beginning and end of a whole nystagmus oscillation.

##### 2.3.1.3 Intensity

Intensity, I is defined as the product of frequency and amplitude (Equation 3)

(3) I=A×f

where A is the amplitude and f is the frequency. The motivation behind intensity is to quantify the “amount” of nystagmus in a gaze test, in order to identify “dominant” and “weaker” nystagmus in eccentric gaze tests. This becomes particularly relevant when dealing with patients with peripheral nystagmus which follows Alexander’s law. Alexander’s law refers to a pattern of behaviour, where when looking in the direction of the fast phase the intensity of nystagmus increases, similarly decreasing when looking in the opposite direction of the fast phase. Figure 2 shows a clear example of this as the intensity of the nystagmus reduces significantly going from the left gaze test to primary and right gaze tests. This behaviour is typical of patients with peripheral lesions and is caused by a superposition of multiple effects which can either combine constructively to increase the nystagmus intensity, or combine destructively to decrease the intensity (8, 17).

Thus, due to Alexander’s law if two patients have peripheral lesions but in opposite affected ears then it is inappropriate to compare the two right gaze tests. It would be more appropriate to compare the gaze tests in direction of each patient’s lesion. This is where the intensity feature can be used to separate the dominant gaze direction with higher intensity oscillation (i.e., in the direction of the lesion for peripheral patients) from the weaker side with lower intensity oscillations. Differentiating dominant and weaker gaze tests is central to the development and analysis of features and biomarkers. Henceforth, to differentiate features from the dominant and weaker eccentric tests subscripts d and w are used, such that in all cases $I_{d}>I_{w}$ . Intensity is calculated three times using the average, minimum, and maximum measures of amplitude and frequency.

##### 2.3.1.4 Frequency-amplitude

Similarly to intensity, the frequency-amplitude ratio, FA , is a feature derived from frequency and amplitude, and is defined in Equation 4

(4) FA=fA

It characterizes the contribution of frequency and amplitude to the intensity of the signal. Hence differentiating between high amplitude/low frequency and low amplitude/high frequency signals. There are no studies evaluating the differences in this ratio between peripheral and central nystagmus. The expected behaviour is that peripheral lesions will have lower frequency and higher amplitude compared to central lesions, particularly on the dominant gaze test (8). The FA ratio is calculated three times using the average, minimum, and maximum measures of amplitude and frequency.

##### 2.3.1.5 Slow & fast phase velocity

In the literature, the feature most commonly seen extracted from nystagmus time series is the slow phase velocity ( $v_{SP}$ ) (9–12). This motivated the use of this feature as a “state-of-the art” baseline differentiator between peripheral and central lesions, and its expansion the fast phase $v_{FP}$ . More accurately, in this paper, the slow and fast phase velocities refer to the absolute value of the average velocity over a slow or fast phase, as shown in Equation 5

(5) $$v_{SP}=\frac{\left|r_{\;1}-r_{\;0}\right|}{t_{1}-t_{0}}\;\mathrm{and}\;v_{FP}=\frac{\left|r_{\;2}-r_{\;1}\right|}{t_{2}-t_{1}}$$

where $r_{i}$ and $t_{i}$ refers to the eye rotation and time at increment i , with i=0,1 at the start and end of the slow phase and i=1,2 at the start and end of the fast phase. The slow and fast phase velocities were estimated using the segmented positional data, by dividing the position delta by time delta as shown in the equations. Average velocity is selected as it reduces the sensitivity of the features to noise; unfortunately, this also decreases the sensitivity to peak velocities in the slow phases, which are expected to be higher in central lesions due to the exponential decay shape of the slow phase (8). The absolute value is included to remove the impact of the direction of the nystagmus. Only two measures, average and standard deviation, are used to summarise the slow and fast phase velocities; however, the ratio of average velocities is also recorded.

##### 2.3.1.6 Slow & fast phase periods

Defined as the amount of time from the start to the end of each phase, slow and fast phase periods ( $T_{SP}$ , $T_{FP}$ ) are expected to have a strong inverse correlation with frequency. This is particularly true for the slow phase, as it can be considered a proxy for the period of a nystagmus oscillation, since the eye is in slow phase for the majority of the oscillation. As with velocity, the mean and standard deviation is recorded for each gaze test along with the ratio of average periods.

#### 2.3.2 Biomarkers

In contrast to the features where each gaze tests is analysed independently, biomarkers draw information from multiple gaze tests. The two biomarkers presented below, directionality and intensity differential are based on physiological principles known to differentiate central and peripheral lesions.

##### 2.3.2.1 Directionality

The directionality biomarker, D , evaluates changes in direction when looking in horizontally eccentric gaze directions (i.e., left and right gaze tests). In most cases patients with a central lesion will have “gaze-evoked” nystagmus, where the direction of the gaze determines the direction of the fast phase (see Figure 1). This is because central nystagmus is caused by an inability to maintain gaze in the eccentric position due to a “leaky” neural integrator (8, 18). This contrasts spontaneous nystagmus—typical of peripheral lesions—which is unidirectional and triggered by a persistent asymmetry in the firing rates of the vestibular nerves (19). Directionality is a binary variable defined by Equation 6

(6) $$D=\{\begin{array}{c} 1\;\mathrm{if}\;\mathrm{no}\;\mathrm{change in nystagmus direction}\\ -1\;\mathrm{if change in nystagmus direction} \end{array}$$

where the direction of the fast phase, defined as $r_{2}-r_{1}$ (i.e., amplitude without absolute value), is used as a reference to calculate direction changes. The directionality biomarkers is expected to be positive in unidirectional spontaneous nystagmus, and negative in gaze-evoked nystagmus. Spontaneous and gaze-evoked nystagmus are heavily correlated with peripheral and central lesions respectively, and so directionality is expected to be an effective separator. However, central lesions can also present with spontaneous nystagmus and in these situations, other ocular motor or neurological features (e.g., head impulse test, severe truncal ataxia etc.) may be required to better identify the aetiology. Directionality is calculated three times using the average, minimum, and maximum measures of $r_{2}-r_{1}$ .

##### 2.3.2.2 Intensity differential

The intensity differential biomarker, $I_{\mathrm{diff}}$ , evaluates variations in intensity between horizontally eccentric gaze tests. This biomarker is inspired by Alexander’s law, however removes the direction element—instead focusing purely on the nystagmus intensity of the eccentric gaze tests. The hypothesis being that a peripheral lesion, which obeys Alexander’s law, will have a greater delta of nystagmus intensity between dominant and weaker gaze tests than a central lesion (20). Although it is noted that intensity changes in the nystagmus can also be present in patients with central lesions (e.g., Bruns nystagmus), and in such cases this biomarker alone may be insufficient. Two versions of the biomarkers are proposed below, a difference (Equation 7) and ratio (Equation 8) of the dominant and weaker sides’ intensities:

(7) $$I_{\mathrm{diff}\left(v1\right)}=I_{d}-I_{w}$$

(8) Idiffv2=IdIw

Given the change in intensity is hypothesized to be noticeably larger in peripheral cases than in central cases, in both the proposed formulas, peripheral lesions are expected to have larger values than central lesions. The ratio version, $I_{\mathrm{diff}\left(v2\right)}$ , was defined as above to set a lower bound of 1 and remove the upper bound. Given $I_{d}\geq I_{w}$ , the reciprocal (i.e., $I_{w}/I_{d}$ ) limits the distributions between 0 and 1, potentially impeding the biomarker’s ability to differentiate between central and peripheral lesions. AL is calculated three times using the average, minimum, and maximum measures of intensity.

### 2.4 Evaluation metrics

In order to measure the biomarkers’ and features’ efficacy, we first calculate the area under the receiver operating characteristic curve (AUC ROC). AUC ROC is a classification metric which measures the performance of a variable across a range of thresholds. Using the receiver operator curve an optimal threshold to separate classes can be established using the Youden threshold. Using the optimal threshold we find the accuracy, positive predictive value (PPV), and negative predictive value (NPV) of each feature or biomarker.

### 2.5 Support vector machine

The gaze test features are also used to develop a support vector machine (SVM) with a linear kernel used as a simple machine learning model to benchmark the performance of the biomarkers. Given the 72 total features (24 per gaze test) and only 24 samples, we select the two best features per gaze test which are not strongly correlated to each other to avoid overfitting; leaving the SVM with six input features. The limited number of samples in our dataset prevents the typical use of training, validation, and testing datasets—instead we use the leave-one-out training scheme. This method entails the training of n models for a dataset of n samples where each model is trained using all but one sample. The sample which is “left-out” of the training set is used as a test sample.

The model uses the squared-hinge loss and a L1 regularization in order to minimise coefficients of the less relevant input features as efficiently as possible. The accuracy of the model is estimated by calculating classification metrics (accuracy, PPV, and NPV) across all *n* models and test sets.

## 3 Results

### 3.1 Segmentation

**Figure 5:**
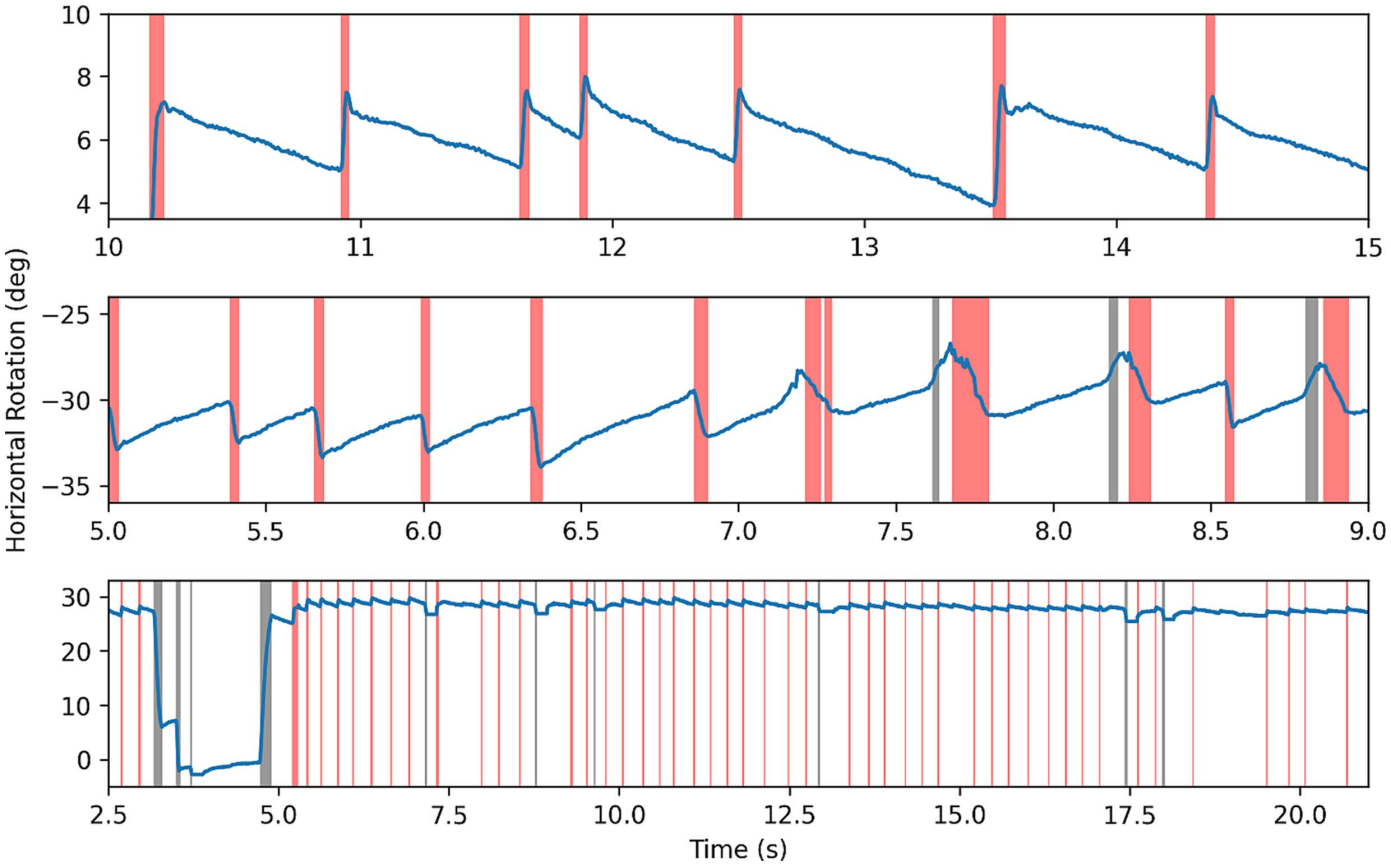
Results of segmentation algorithm on example nystagmus traces. Red highlighting for fast phases, black for other saccades or noise, and blank for slow phase.

Segmentation of some nystagmus time series can be seen in Figure 5—fast phases highlighted in red, erroneous saccades in black, and slow phases left blank. In data with clear nystagmus, the segmentation algorithm was effective, accurately locating transitions between phases despite at times strong noise. The algorithm was able to deal with erroneous saccades well, although struggling with smaller blinking saccades in the same direction as fast phases.

### 3.2 Features

The results of classification metrics of the best six features per gaze test are shown in Table 2. Eighteen of 72 (25%) tested features have a classification accuracy above 70%. The primary gaze test was the best at differentiating between cohorts producing 11 features with an accuracy above 70%, and the features with the highest accuracy (82.6%), PPV (1.0), and NPV (1.0). The most prominent features relate to the phase velocities and periods. Of particular interest is the slow phase period standard deviation which is the overall best performing feature with the best AUC ROC, highest accuracy, and a balanced performance across both classes (PPV of 0.833, NPV of 0.818). As inputs to the SVM we chose the standard deviation of slow phase periods and fast phase period from the primary gaze test (marked with * in Table 2).

**Table 2:** Summary of classification performance of features (PPV, positive predictive value; NPV, negative predictive value).

Gaze test	Feature	Accuracy (%)	PPV	NPV	AUC ROC
**Dominant eccentric**					
	FP period*	78.3	0.667	0.909	0.788
	Amp (max)	73.9	0.500	**1.0**	0.742
	FA (max)*	73.9	0.833	0.636	0.735
	Amp (std)	73.9	0.500	**1.0**	0.674
	SP vel (std)	73.9	0.833	0.636	0.644
	SP period (std)	69.6	0.750	0.636	0.659
**Weaker eccentric**					
	I (min)*	78.3	0.833	0.727	0.788
	Freq (min)	73.9	0.917	0.545	0.788
	Amp (min)	69.6	0.833	0.545	0.682
	SP period*	69.6	0.500	0.909	0.667
	SP period (std)	69.6	0.583	0.818	0.659
	Freq (std)	69.6	0.750	0.636	0.523
**Primary**					
	SP period (std)*	**82.6**	0.833	0.818	**0.871**
	FP period*	**82.6**	**1.0**	0.636	0.780
	SP-FP vel ratio	78.3	0.583	**1.0**	0.833
	SP vel	78.3	0.667	0.909	0.811
	SP-FP period ratio	78.3	0.583	**1.0**	0.795
	Freq (min)	78.3	0.583	**1.0**	0.780

*Indicates features selected as inputs to the support vector machine. Bold values show highest scores.

While the accuracies and AUC ROC values are similar between dominant and weaker gaze test features, the most informative features from dominant gaze tests relate to fast phase characteristics (i.e., period, amplitude, FA ratio) and to intensity and time (i.e., frequency and phase periods) for weaker gaze tests. Hence, for the SVM we chose the fast phase period and FA ratio as input variables from the dominant gaze test, and intensity and slow phase period from the weaker gaze test (marked with * in Table 2). Furthermore, the dominant gaze test features tend to have higher NPVs and lower PPVs compared to the weaker gaze test features—suggesting that the dominant gaze test is better at identifying peripheral lesions and the weaker is better at identifying central lesions.

**Figure 6:**
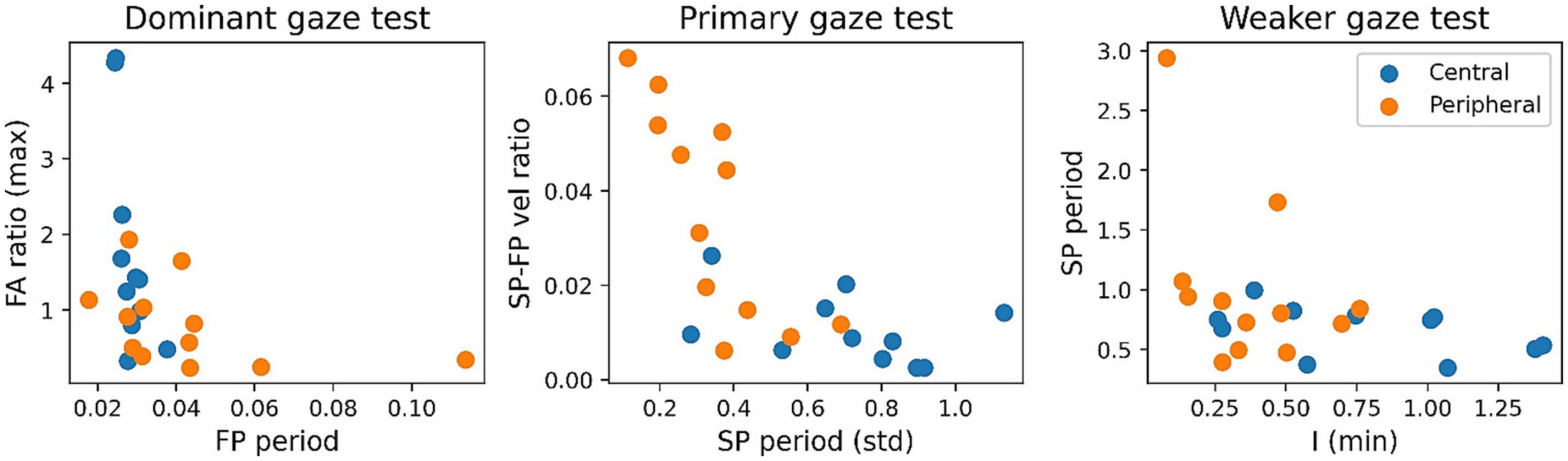
Scatter plots of selected SVM features by gaze test, colour coded by patient cohort.

For the SVM the selected input features defined above are plotted against each other in Figure 6, despite selecting the features which are performant and uncorrelated, the classes have poor separability. This translates to the performance of the SVM which is unable to outperform the individual features with an accuracy of 75.0%, PPV of 0.8 and NPV of 0.714 (see Table 3). Interestingly, the average coefficients for all input features show that the SVM model most heavily relies on the SP-FP velocity ratio in the primary gaze test (coefficient of −0.63), the fast phase period of the dominant gaze test (coefficient of −0.26), and the intensity feature from the weaker gaze test (coefficient of 0.21) to classify the samples, with the other three features all have coefficients of magnitude below 0.1.

**Table 3:** Summary of classification performance of biomarkers and SVM model (PPV, positive predictive value; NPV, negative predictive value).

Biomarker	Accuracy (%)	PPV	NPV	AUC ROC
Directionality (avg)	100	1.0	1.0	—
Directionality (min)	100	1.0	1.0	—
Directionality (max)	100	1.0	1.0	—
Intensity diff. − v1 (max)	**79.2**	**0.667**	0.917	0.750
Intensity diff. − v1 (avg)	75.0	0.500	**1.0**	**0.778**
Intensity diff. − v2 (avg)	75.0	0.583	0.917	0.750
Intensity diff. − v2 (max)	75.0	**0.667**	0.833	0.736
Intensity diff. − v2 (min)	70.8	0.500	0.917	0.701
Intensity diff. − v1 (min)	62.5	**0.667**	0.569	0.569
SVM model	75.0	0.800	0.714	—

Bold values show highest scores.

### 3.3 Biomarkers

As shown in Table 3, the directionality biomarker is a particularly strong differentiator, achieving 100% accuracy and 1.0 AUC ROC on this dataset irrespective of the metric—average, minimum, maximum—used to calculate changes in direction. While the performance of $I_{\mathrm{diff}}$ is distinctly worse, it still matches the performance of the best individual gaze test features (four rows in Table 3 have accuracies above 75%). Of the two versions of the intensity biomarker, $I_{\mathrm{diff}\left(v1\right)}$ (difference of intensities) is generally better than $I_{\mathrm{diff}\left(v2\right)}$ (ratio of intensities). Surprisingly, $I_{\mathrm{diff}}$ struggles to identify central patients with a max PPV of 0.667, compared to the highest NPV of 1.0. From the results, the best intensity differential biomarker is $I_{\mathrm{diff}\left(v1\right)}$ using the maximum amplitude and frequency features having an accuracy if 79.2%, although its performance between classes is unbalanced with an NPV of 0.917 and PPV of 0.667.

**Figure 7:**
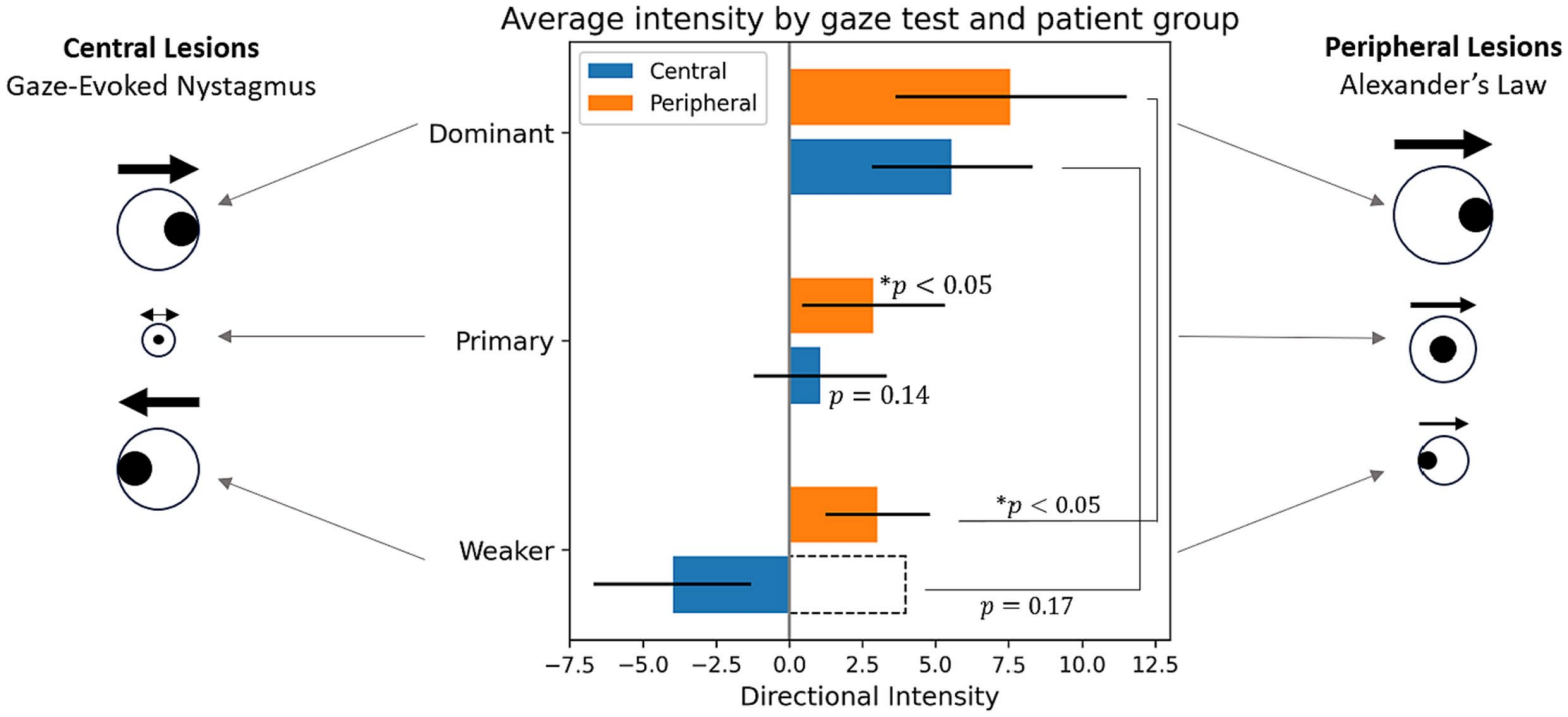
Average directional intensities across gaze direction by patient cohort. The sign of directional intensity refers to direction, where positive is the direction of the dominant gaze test, and the magnitude is the same as intensity feature ( I=A×f ). For statistical tests intensity was used rather than its directional counterpart as indicated by the dotted bar outline. *Indicates statistical difference to a zero distribution or between two sets of intensities.

The choice of biomarkers is validated by Figure 7, which illustrates nystagmus behavioral patterns across the dataset by plotting the average intensity in each gaze test by patient cohort. The figure confirms the results of the classification metrics—for all central patients, there is a change in nystagmus direction, seen in the “negative” directional intensity of the weaker gaze tests. It also supports the initial hypothesis of $I_{\mathrm{diff}}$ , with significant changes in intensity between eccentric gaze tests for peripheral patients (*p* < 0.05), but none for central patients (*p* = 0.17).

## 4 Discussion

This work aimed to present quantitative nystagmus biomarkers which reflect known clinical assessments used by specialists to determine the origin of AVS. In order to test the biomarkers, a pipeline starting from eye movement time series to the robust analysis of the performance was presented. The results are encouraging although limitations remain—particularly with regards to the limited size of the dataset and effect this may have on the pipeline’s generalizability.

### 4.1 Segmentation

The segmentation algorithm was robust and the use of dynamic heuristic thresholds to adapt to individual gaze tests proved to be an effective method to deal with the natural variability in clear nystagmus signals. However, in cases of small amplitude nystagmus the algorithm was inconsistent, at times missing nystagmus oscillations when trying to avoid noise. Furthermore, as the algorithm assumes the presence of nystagmus, some errors arose in its absence. For instance, the segmentation algorithm occasionally correctly identified a lack nystagmus but incorrectly labeled blinking saccades as nystagmus oscillations. While this is not an issue in strong signals with the nystagmus behaviour dominating the features, in weaker signals the blinking artifacts dominate output features. Alternatively, random small eye movements are identified as fast phases, leading to particularly unpredictable results.

### 4.2 Features

A prominent trend emerging from the feature testing is that the primary gaze test is the best at differentiating central and peripheral lesions. While this was not anticipated, a possible explanation is that in this dataset, nystagmus is minimal from the primary gaze test of gaze-evoked nystagmus caused by central lesions. This contrasts to peripheral lesions which tend to have clear nystagmus in the primary gaze test. This is further confirmed by independent *t*-tests in which the primary gaze test intensities of peripheral patients were found to be statistically different from a zero distribution (*p* < 0.05) and those of the central patients were not (*p* = 0.14; Figure 7). This causes the primary gaze test to be the gaze direction with the most pronounced differences between cohorts.

In the weaker eccentric gaze test, significant features were frequency and intensity, with slow phase velocity also prominent. This can be explained by peripheral nystagmus manifesting as a low frequency and small amplitude “spontaneous” nystagmus due to tonal vestibular imbalance, rather than a “gaze-evoked” central nystagmus that is induced with gaze. In the dominant eccentric gaze tests key features were amplitude, fast phase period, and frequency-amplitude (FA) ratio. This is in line with expected behaviour and is attributed to Alexander’s law causing peripheral lesions to trigger lower frequency larger amplitude nystagmus oscillations compared to their central counterparts.

The SVM failed to outperform the individual features. However, even though the accuracy was low, the models’ coefficients were consistent across the leave-one-out models which suggests selected features are robust to inter-patient variability. It is important to note that, some features selected as inputs to the SVM have little-to-no biological reasoning behind them—they are selected purely based on their ability to differentiate the classes. This data driven approach to classification is representative of typical “black-box” models, and contrasts to the clinically-inspired biomarkers. The model’s low accuracy can be attributed to two issues; firstly the poor separability of the input features made it difficult for the SVM to locate boundaries between classes. Secondly, the limited dataset restricted the modelling to a linear kernel, more complex kernels were tested, but deemed inappropriate as they overfit during training and performed worse on the test set. An increased dataset with some improvements to the data pipeline could allow for improved features selection with increased interclass variation while reducing intraclass variation.

### 4.3 Biomarkers

Focusing on the novel quantitative biomarkers, the directionality biomarker performed particularly well, being able to systematically differentiate between gaze-evoked and spontaneous nystagmus, and hence the peripheral and central cases present in our dataset. However, in a systemic review which included 239 central cases of acute vertigo, only 38% presented with direction-changing nystagmus (21), highlighting that a directional biomarker alone is insufficient to discriminate central from peripheral cases of vertigo. The $I_{\mathrm{diff}}$ biomarker also performed well, with accuracy and AUC ROC measures comparable to the performance of the best single gaze test features. It is thought that biomarkers were able to generally outperform the features due to the their robust biological reasoning and greater “access” to information by combining features from multiple gaze tests.

### 4.4 Limitations

The primary limitation inherent to the segmentation algorithm is the fundamental assumption that nystagmus is present. A possible mitigation is the integration of a classifier which confirms the presence of nystagmus before segmentation. The algorithm is further limited by its struggles with small amplitude nystagmus in noisy signals. While the segmentation struggles impacts all downstream processing, the intensity differential biomarker is particularly affected. As improvement in measurements of small amplitude nystagmus are expected to translate directly to improvement in its performance.

It is also important to emphasise the limited size and homogeneity of the dataset. These results are preliminary and the classification metrics remain vulnerable to significant changes upon the inclusion of a larger and more diverse patient population. This caveat underscores the need to expand the size of the dataset and verify the generalizability of our results. However, given the biomarkers are based on clinically validated nystagmus characteristics there is strong cause to expect these results will generalise effectively to a broader dataset; comparing favourably to SVMs and other machine learning techniques which rely statistical trends found in larger datasets.

## 5 Conclusion

In conclusion, these results are highly encouraging and strongly motivate further investigation into nystagmus biomarkers, such as vertical nystagmus, up and down gaze tests, and wave-shapes which are known to be diagnostically relevant (8, 22, 23). While acknowledging the limitations of our small sample size, these preliminary findings suggest significant potential, with the biomarkers surpassing the performance of features conventionally used in nystagmus analysis, and a baseline machine learning model. Furthermore, since our biomarkers derive from clinically validated characteristics of nystagmus, they hold a fundamental advantage over any other quantitative measure or model. Precise, quantitative, serial eye movements assessment is recommended in patients with AVS given evolution of clinical signs within the first 24 h (24) so repeated evaluation using AI may shed further diagnostic information in this setting. Such a marriage of human and artificial intelligence maybe the way forward to reduce burden of interpretation placed on point-of-care physicians and bridging the knowledge gap to specialists to the direct benefit of all patients.

## Data availability statement

The original contributions presented in the study are included in the article/supplementary material, further inquiries can be directed to the corresponding author.

## Ethics statement

Ethical approval was obtained from the United Kingdom Northwest-Greater Manchester South Research Ethics Committee (Approval No. 21/ NW/0015). The studies were conducted in accordance with the local legislation and institutional requirements. The participants provided their written informed consent to participate in this study.

## Author contributions

BD: Data curation, Formal analysis, Investigation, Methodology, Visualization, Writing – original draft, Writing – review & editing. TL: Investigation, Methodology, Software, Supervision, Validation, Visualization, Writing – review & editing. NK: Data curation, Supervision, Writing – review & editing. DK: Conceptualization, Data curation, Investigation, Project administration, Visualization, Writing – review & editing.
